# The role of labeled cell therapy with and without scaffold in early excision burn wounds in a rat animal model 

**DOI:** 10.22038/ijbms.2020.34324.8156

**Published:** 2020-05

**Authors:** Seyedeh Sara Hashemi, Mohammad Reza Pourfath, Amin Derakhshanfar, Abbas Behzad-Behbahani, Javad Moayedi

**Affiliations:** 1 Burn and Wound Healing Research Center, Shiraz University of Medical Science, Shiraz, Iran; 2 Nour Danesh Institute of Higher Education, Isfahan, Iran; 3 Diagnostic Laboratory Sciences and Technology Research Center, School of Paramedical Sciences, Shiraz University of Medical Sciences, Shiraz, Iran; 4 Center of Comparative and Experimental Medicine, Shiraz University of Medical Sciences, Shiraz, Iran

**Keywords:** HWJMSCs, Labeled cells, Wound healing, 2-dimensional cell culture, 3-dimensional cell culture

## Abstract

**Objective(s):**

One of the essential problems in burn therapy is performing the permanent replacement of skin in full and deep thickness injuries. Human Wharton’s Jelly mesenchymal stem cells (HWJMSCs) have a unique combination of prenatal and postnatal properties. Decellularized human amniotic membrane (DHAM) can be used as a scaffold for HWJMSCs-therapy. We aimed to evaluate the quantity and quality of healing in the early excision burn wound dressing with 3-dimensional and 2- dimensional cell cultures.

**Materials and Methods:**

Amniotic and umbilical cords were isolated from the mothers who were candidates for cesarean section. HAM was decellularized using the mechanical and enzymatic method. HWJMSCs were isolated and cultured; cell surface markers were examined for authentication of MSCs and labeled using a viral vector containing the *cGFP* gene. Burns were created using brass bar in 32 adult male Albino rats and randomly divided into four groups (DHAM+HWJMSCs, injection of HWJMSCs, HWJMSCs was spread on the wound, and DHAM alone). Rats were sacrificed on the 7^th^ and 14^th^ days for pathological examination of the wound. Comparisons between the study groups were made by one-way analysis of variance.

**Results:**

Wound healing process in DHAM+HWJMSCs was much more progressed during the first week in comparison to other groups, and exhibited significant differences in re-epithelialization, formation of granulation tissue, and hemorrhage (*P*<0.05).

**Conclusion:**

The utility of the amniotic scaffold seeded by the human mesenchymal stem cells is recommended for accelerating the healing process.

## Introduction

Severe burns are considered a life-threatening factor in developing countries. Burn patient’s treatment prolongation may lead to incurable infection and septic shock (1). Great efforts have been made to reduce the burn wound treatment period. Autograft has been used as the best method to treat severe wounds and burns; however, it has its own drawbacks including ulceration of the skin in the area to be harvested and transplant rejection. In addition, for those with burns up to 50% of total body surface area (TBSA) or more, the target areas for harvesting the skin are limited (2).

Tissue engineering has emerged as an interdisciplinary science in order to create biomaterials to be used as a replacement for damaged skin or organs. In the case of skin, the ultimate goal is to produce a structure that accelerates active skin formation. This is not possible unless we provide cells with appropriate resources and a good scaffold (3). Hence, one of the most important roles of tissue engineering is to provide a suitable scaffold for cells to grow. Scaffolding should be able to integrate into the host’s skin easily and generate a suitable environment for cell growth. Currently, several materials have been examined to make the skin matrix, including collagen-elastin membrane, acellular cadaveric dermis, collagen-glycosaminoglycan membranes (GAG), plasma and hyaluronic acid, etc. (4-7).

Human amniotic membrane (HAM) is considered an important source of cellular scaffolding due to its structure, biological characteristics, mechanical properties, and low immunogenicity. HAM is the innermost layer of the placenta which consists of several parts: (1) one layer of cubic cells; (2) basement membrane derived from skin that contains laminin 5 and collagen types IV, VII, XVII; (3) a cell-free layer; and (4) the underlying fibroblast and sponge layers. There are no blood vessels or nerves in HAM (8). During the treatment process, HAM is applied to cover partial-thickness wounds, which is comparable with donor tissue. Additionally, HAM can reduce pain and inflammation in burned areas, accelerates epithelialization, inhibits scar creation, and prevents infection, all due to its good adhesion characteristics (9). In addition, HAM exhibits some suitable physical characteristics such as permeability, durability, elasticity, flexibility, and plasticity (8).

Mesenchymal stem cells (MSCs) have become popular as a solution to wound healing. Some other feasible healing mechanisms of MSCs include secretion of paracrine signals, as an example for the release of hematopoietic factors, not inciting the immune system, applying stem cells found in the tissue to the differentiation of other tissue cells. Amongst various sources of MSCs, the umbilical cord is accepted as a universal source of MSCs due to its constant availability and cost-effectiveness. The umbilical cord MSCs have been shown to have some advantages over bone marrow and fat MSCs. MSCs isolated from the Wharton’s Jelly (WJMSC) were proven to have a wide source of embryonic, non-immunogenic, and non-tumorigenic cells. These cells can be grafted to people for several purposes such as heart, bone, cartilage, fat, pancreas, nerve, skin, blood vessels, and organ transplantation (10). Human WJMSCs reduce pulmonary, kidney, and liver fibrosis (11, 12), and these cells are able to differentiate into gland-like cells that accelerate skin restoration (13).

The current study aimed to investigate the application of HAM as a suitable scaffold to support HWJMSCs used in burn wound healing in a rat model. Considering the results of previous studies, the combination of these two methods seems to speed up the healing process that might establish a new therapeutic procedure in order to reduce the treatment period of severe burn wounds.

## Materials and Methods

***Preparation of amnion and umbilical cord***

Amnion and umbilical cords were collected from selected cesarean sections in the 36^th^ and 37^th^ weeks of pregnancy at Ghadir Mother and Child Hospital (Shiraz, Iran). Written informed consent was obtained from all subjects for the collection of amnion and umbilical cords for research purposes. All donor mothers were tested for hepatitis B virus (HBV), hepatitis C virus (HCV), human immunodeficiency virus (HIV), and Syphilis. Although umbilical cord and amnion are disposable tissues, parents were informed about our research and they were asked to fill in the consent forms. After the surgery, all tissues were treated by PBS containing 50 μg/ml of streptomycin and 50 μg/ml of penicillin in aseptic conditions and were taken to the laboratory at 4 °C. All of the steps in the laboratory were performed in a sterile environment and under the cultural hood. The amniotic membrane was separated from the chorionic membrane by a mechanical method and peeled by cold PBS (4 ^°^C) to remove the blood (14). This study was approved by the local Ethics Committee of Shiraz University of Medical Sciences, Shiraz, Iran (June 2, 2015, approval no. IR.SUMS.REC.1394.S172).

***Wharton’s jelly isolation, culture, and labeling***

The umbilical cords were washed with phosphate buffered saline (PBS, pH = 7.2) to remove the blood, minced into 2-mm^2^ pieces, and transferred to 10-cm^2 ^culture plates containing DMEM/F12 (catalog No. 11320033, Gibco-Invitrogen, USA) supplemented with 10% fetal bovine serum (FBS) (catalog No. 26140079, Gibco-Invitrogen, USA), penicillin-streptomycin solution (100 μg/ml) (catalog No. P4333, Sigma-Aldrich, UK). When the cell confluence reached 70–80%, cells were passaged by trypsin/EDTA 0.25% solution (catalog No. T4049, Sigma-Aldrich, UK). Stem cell potency of HWJMSCs was examined after the third passage by flow cytometry analysis. Suspension of the cells in 50 μl sterile PBS with 10 μl conjugated antibodies, FITC labeled CD105 and CD34 (Sigma, St. Louis, MO, USA), was verified by mesenchymal surface markers with at least 1000 events being analyzed. Afterward, the cells were transduced with lenti-cGFP (a gift from Trono lab, EPFL University, Lausanne, Switzerland) at a multiplicity of infection (MOI) of 16904. After transduction, the cells were exposed to 2 μg/ml puromycin (catalog No. P9620, Sigma-Aldrich, UK) for 2 days to obtain the stable transduction (15).

***Amnion 3D culture and MTT assay***

Collected amnion after preparation and without decellularization was placed in the bottom of the plate in such a way that the epithelial layer faced upside (16). The HWJMSCs which came from 3^rd^ passage were treated by a lentiviral vector including the cGFP gene to make its tracking possible in the next *in vivo* steps. Approximately, 1×10^6^ HWJMSCs which were suspended in DMEM/F12 and supplemented with 10% FBS, were seeded onto the epithelial surface of acellular amniotic membrane. The harvested suspension, which contained Wharton’s jelly stem cells and acellular amnion, was placed in culture plates in a humidified incubator in a 5% CO_2_ atmosphere for 24 hr. Amnion scaffold cell viability was evaluated using the MTT assay (17, 18). Each experiment was repeated at least three times and the main results were obtained as the mean of each 3 test results.

***In vivo*** ***rat model***

Thirty-two adult male Albino rats (180–200 g, 6–8 weeks old) were purchased from the Center of Comparative and Experimental Medicine, Shiraz University of Medical Sciences, Shiraz, Iran. All procedures were approved by the committee of animal care at the Shiraz University of Medical Sciences, Shiraz, Iran and conducted in accordance with the established guidelines. During this study, rats were kept one per coop under the retained situation (21±2 ºC, 65–70% of relative humidity, and had free access to water and food).

The rats were subjected to intraperitoneal anesthetic induction with 90 mg/kg ketamine (Alfasan Co., Netherlands) and 9.0 mg/kg xylazine (Alfasan Co., Netherlands) (19). The proximal parts of rat necks were shaved and the skin was scrubbed with povidone iodine solution. Afterward, the skin was wiped with sterile water. For induction of the 3^rd^ degree burns (20% of TBSA with a total involvement of all layers of the skin), the brass bar technique was used (the area of the square-like head of the brass bar was 1.3-cm^2^ capable of reaching 105 ºC and chosen on the basis of the average weight of the rats used) (20). The thermal burn was used to produce the lesion by means of direct contact for 5 sec.

After 24 hr, the necrotic place of wounds with 1 mm of marginal skin was excised out using a scalpel under general anesthesia. To prevent infection, the wounds were disinfected with 70% ethanol (for 1 sec), then washed with normal saline (for 30 sec). At this time, rats were divided into four equal groups (n=8) and the treatment was applied to the wound surface as follows: group 1 was treated with DHAM+HWJMSCs; group 2 was treated with an injection of HWJMSCs; in the group 3, HWJMSCs were spread on the wound; and in group 4, DHAM was used alone. The preparation process of DHAM, HWJMSCs, and DHAM+HWJMSCs was described previously (16). Each treatment was carried out once at 24 hr after burn injury. To prevent dehydration, wounds were sutured with sterile gauze vaseline. On the 7^th^ and 14^th^ days after the creation of burn injuries, half of rats in each group (n=4) were euthanized under standard guidelines of Animal Research Advisory Committee for Rodents Using Carbon Dioxide (21, 22). Imaging and skin wound biopsy were performed. The specimens were fixed in 10% buffered formalin and then embedded in paraffin. Paraffin blocks were cut into 5 mm pieces and stained using Hematoxylin and Eosin (H & E) (Merck, Germany) (23).

***Statistical analysis***

Statistical analysis was conducted using the Statistical Package for Social Sciences software package (IBM SPSS, version 21). All values were expressed as means and standard deviations (SD). Comparisons between the study groups were made using one-way analysis of variance (ANOVA) with a *post-hoc* Tukey’s test. The Pearson’s χ2 test was used for assessing differences between categorical variables. A *P-value* less than 0.05 was considered as statistically significant.

## Results

***HWJMSCs expansion and amnion 3d culture***

**Figure 1.**
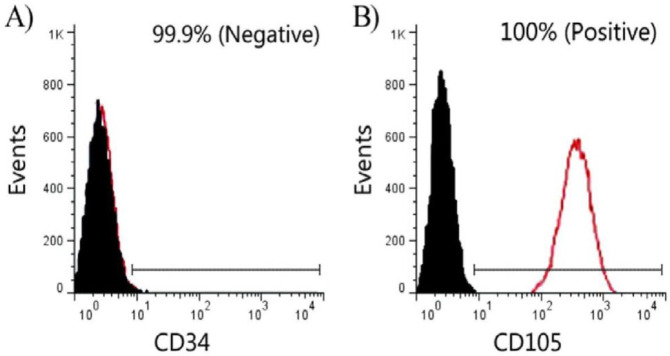
Flow cytometry analysis for Human Wharton’s Jelly surface markers: A: 99.9% negative for CD34; B: 100% positive for CD105

**Figure 2:**
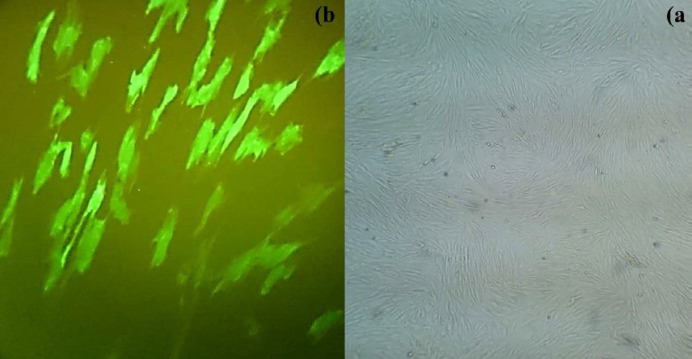
Human Wharton’s Jelly mesenchymal stem cells transduced with lentiviral vectors. The same region, A: under a light microscope (x4); and B: under UV (x10)

According to safety protocols in the extraction of the amniotic and umbilical cords, each of the two mothers participating in our study was evaluated for sexually transmitted diseases, including HIV, Hepatitis B, C, and Syphilis, and the results were found to be negative. After separation and reaching the third cell passage, HWJMSCs were examined for cell surface factors (CD105 and CD34) (Figure 1). Flow cytometry analysis for CD105 and CD34 showed 100% positive and 99.9% negative results, respectively. These phenotypes confirmed that isolated cells are HWJMSCs (24). The 293LTV cell line was purchased from Iran’s Pasteur Institute (Tehran, Iran) and co-transfected by the lentiviral expression (pPCDH-cGFP), packaging (psPAX2) and envelope (pPMD2.G) plasmids. The expression of cGFP was confirmed by fluorescence microscopy. The HWMSCs were transduced using the lentiviral particles carrying cGFP. Finally, clusters of HWMSCs were observed and used for subsequent applications (Figure 2).

***MTT assay***

**Figure 3:**
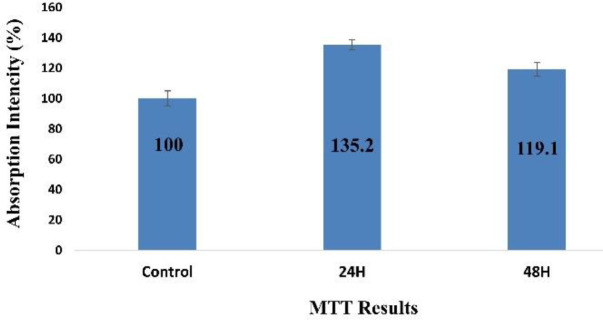
Evaluating the viability of HWJMSCs on decellularized human amniotic membrane: MTT results show that DHAM enhances cell viability nearly 1.4 times after 24 hr (*P*<0.0001), and 1.2 times after 48 hr (*P*=0.001), respectively

To investigate the effects of HWJMSCs on acellular HAM, the viability of cells after labeling by Gene cGFP [Figure 2] and perching on DHAM was evaluated using MTT assay. The statistical results for these cells were compared with the controls. HWJMSCs represent an increase in the viability of the cells on the amniotic scaffold after 24 hr (*P*<0.0001) and 48 hr (*P*=0.001) (Figure 3).

***In vivo tracking of HWMSCs in tissues excised out of the burned area***

In order to corroborate the contribution of HWMSCs to the wound healing process, their presence in the burned area was further confirmed in tissues excised out of such regions (emission = 535 nm and excitation = 470 nm). At the end of the first and second weeks after treatments, the tissue biopsies were collected from the burned areas of the treated and untreated animals. The cGFP-expressing HWMSCs were detected in the tissues obtained from the burned area as patches of cells. On the other hand, no cGFP signal was observed in control rats that had not received HWMSCs and other vital organs from both treated and untreated rats.

***Histopathological findings***

Wound healing processes initiated during the first 7 days. At this time, early re-epithelialization (1^+^) was seen in all groups, except in group 3. In addition, early granulation tissue (1^+^) was observed in all groups, similarly, and mild hemorrhage (1^+^) occurred in all groups, except groups 1 and 4, which were affected by moderate hemorrhage (2^+^). Furthermore, mild inflammation (1^+^) was observed merely in group 4.

**Figure 4:**
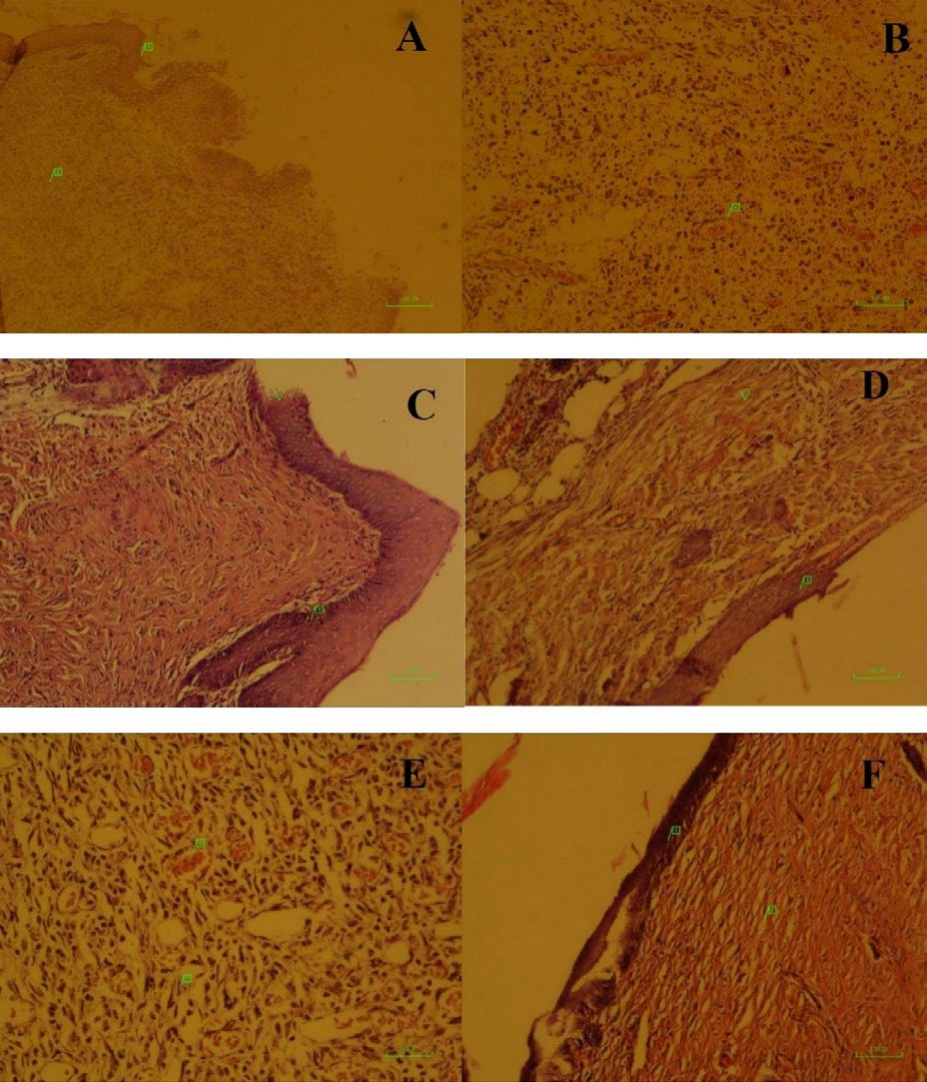
Histological assessment of wound healing after burn injury

On the 14^th ^day, the healing rate in group 1 (DHAM+HWJMSCs) surpassed the other groups. At this time, re-epithelialization was the same (1^+^) in all groups, except in group 1, in which much more re-epithelialization (2^+^) was formed. The formation of granulation tissue was moderate (2^+^) in all groups, except in group 1, in which mature scar (3^+^) appeared. Additionally, mild hemorrhage (1^+^) was seen in all groups, except in group 1. Finally, none of the study groups showed evidence of inflammation at the end of the second week (Table 1, Figure 4).

**Table 1:** Pathological analysis of the wound and wound surrounding cells in the experimental groups. Group 1 was treated with human amniotic membrane covered by HWJMSCs, Group 2 was treated with the injection of HWJMSCs, Group 3 was treated with the spread of HWJMSCs, and Group 4 was treated with the human amniotic membrane on the wound at 7^th^ and 14^th^ days after burn wound excision

**Histopathological findings**				**Study groups**	**Time point**
**Inflammation**	**Hemorrhage**	**Granulation tissue**	**Re-epithelialization**		
Absence	Moderate	Early	Early	**1**	**7** ^th^ ** day**
Absence	Mild	Early	Early	**2**	
Absence	Mild	Early	Absence	**3**	
Mild	Moderate	Early	Early	**4**	
Absence	Absence	Mature	Incomplete	**1**	**14** ^th^ ** day**
Absence	Mild	Immature	Early	**2**	
Absence	Mild	Immature	Early	**3**	
Absence	Mild	Immature	Early	**4**	

A and B treated with the human amniotic membrane on the wound at day 7

A: Incomplete re-epithelialization (1) and early granulation tissue (2) is seen. Inflammatory cells scattered throughout the lesion (40, H&E).

B: Early granulation tissue is characterized by neovascularization (1). Also, inflammatory cells and hemorrhage are seen (x100, H&E).

C (the spread of HWJMSCs, 14th day): Re-epithelialization (arrow head) is begun. The newly formed epidermis is acanthotic (1). More granulation tissue is seen beneath the epidermis (x100, H&E)

D and E treated with the injection of HWJMSCs on the wound at 7^th^ day

D: Re-epithelialization begins but is not completed (1) (x100, H&E)

E: Early granulation tissue is composed of newly formed capillaries (1) and too many fibroblasts (2) (x200, H&E)

F treated with Human amniotic membrane covered by HWJMSCs at the 14th day: Re-epithelialization (1) and scar tissue (2) are seen (x100, H&E)

## Discussion

Nowadays, various types of skin ulcers coverage are being used to treat burn wounds. Selection of appropriate dressing can reduce the severity of the wound, provide complete coverage of the wound, prevent bacterial infections, and promote the ability against mechanical stresses. In patients with burn wounds the frequency of replacing the dressing, moisture keeping quality, and gas exchange quality are also important (25). Our study showed a hastened healing process in rats that had been treated with DHAM+HWJMSCs compared with HWJMSCs rats. The results of the application of adult stem cells in the treatment of burns in several animal models have been indicative of the effective role of these cells in wound healing (26-28). Although allografts, xenografts, skin compounds, and skin analogs are commercially available, it is still necessary to improve the results of skin substitutes. Therefore, tissue engineering should focus on the application of a cover or biological and chemical scaffold that has appropriate characteristics that can create a microenvironment for the stem cell population to make their transfer to wounds possible and efficient (29-31). In fact, autograft in burn patients with a high percentage of burns (more than 60% of TBSA) will actually increase the likelihood of mortality [25]. In many studies, HAM was considered a good biological cover for wound management with rapid wound healing and re-epithelialization ability [27, 28]. Additionally, HAM can be used as a good cover for partial-thickness wounds, as a temporary dressing for freshly excised burn wounds. HAM features include the reduction of the wound severity, prevention of infection, the creation of a stable moist environment to promote healing and efficient wound adhesion. Laboratorial surveys have shown that HAM basement membrane is very similar to the basement membrane of human skin morphologically and ultra-structurally (according to electron microscopy studies) (32). Amnion epithelial sections are known as supporters of proliferation and differentiation of cells associated with the cornea, mucosal cells, and epithelial cells (33). In addition, the amnion stromal sections can represent a significant proliferation and adhesion as skin matrix for fibroblasts cells (34). Limited immunogenicity and anti-inflammatory properties of HAM resulted in its application for the treatment of corneal diseases and even non-human transplantation (35). For HAM to be used as a cell scaffold, at first, the pre-existent cells must be eliminated. Some of these methods are very complicated for use in a laboratory and others failed due to making tissue composition (36, 37). Given that acquiring HAM is easy and its long-term preservation can be accomplished without losing its characteristics, it can be a good scaffold for skin tissue engineering.

In this study, multiple methods each with different success percentages have been proposed. The results indicate the effective role of stem cell placement on the amniotic scaffold compared with non-scaffold stem cells in wound healing. The result of the growth of MSCs on Decellularized HAM (or simply DHAM) indicated that the proper support of these cells and viability rate increased by 135% and 119%, respectively after 24 and 48 hr. Recent evidence suggests that human WJ-MSCs promoted wound healing by paracrine signaling in our culture conditions *in vitro* and in an *in vivo* preclinical animal model. WJ-MSCs represent a feasible, universal, and off-the-shelf technology to enhance normal wound healing and to improve patient survival and quality of life (38).

Several attempts have been made to study HAM anti-infective effects. This seems to be a result of the synthesis of anti-inflammatory proteins and of a reduction of the expression of transforming growth factor-b (TGF-b) and proinflammatory cytokines, such as interleukin-10 (IL-10) (39). A number of studies have shown that HAM helps re-epithelialization and granulation tissue development by inhibiting the activity of white blood cells protease, and it also assists in angiogenesis stimulation (40). With attention to histopathological results, the healing process was much better in the DHAM+HWJMSCs group compared with others. This includes more re-epithelialization, mature and organized scar tissue without any hemorrhage or inflammation. All of the articles included in this study evaluated time to healing using MSCs in wound healing. All of the studies demonstrated accelerated wound closure and enhanced histological parameters in wounds treated with MSC therapies, irrespective of cell isolation or delivery method.

Despite a lack of a standardized histological scale for evaluating cutaneous wound healing, re-epithelialization, granulation tissue, angiogenesis, and inflammation are considered representative features. All studies that reported histological findings identified enhanced wound healing in wounds treated with MSCs compared with control treatment (38, 41-43). Specific features observed included increased recruitment of macrophages, increased angiogenesis (41), and restoration of sebaceous glands and hair follicles (41). A number of studies sought to evaluate the persistence of MSCs in the wound environment after transplantation and demonstrate that MSCs persist in the wound for up to several weeks following transplantation (38, 42, 44). Various mechanisms have been investigated to enhance cell survival following transplantation and typically involve alterations in biomimetic scaffold delivery systems (41).

## Conclusion

Given that acquiring HAM is easy and its long-term preservation can be accomplished without losing its characteristics, it can be a good scaffold for skin tissue engineering. Based on the present investigation, the utility of an amniotic scaffold seeded by human mesenchymal stem cells is recommended for accelerating the healing process after skin burn injuries.
